# Oral Dysbiosis and Neuroinflammation: Implications for Alzheimer’s, Parkinson’s and Mood Disorders

**DOI:** 10.3390/microorganisms14010143

**Published:** 2026-01-08

**Authors:** Laura Carolina Zavala-Medina, Joan Sebastian Salas-Leiva, Carlos Esteban Villegas-Mercado, Juan Antonio Arreguín-Cano, Uriel Soto-Barreras, Sandra Aidé Santana-Delgado, Ana Delia Larrinua-Pacheco, María Fernanda García-Vega, Mercedes Bermúdez

**Affiliations:** 1 Faculty of Dentistry, Autonomous University of Chihuahua, Chihuahua 31000, Mexico; czavala@uach.mx (L.C.Z.-M.); cmercado@uach.mx (C.E.V.-M.); jcanon@uach.mx (J.A.A.-C.); usoto@uach.mx (U.S.-B.); ssantana@uach.mx (S.A.S.-D.); alarrinua@uach.mx (A.D.L.-P.); mfgarcia@uach.mx (M.F.G.-V.); 2 Department of Environment and Energy, Center for Research in Advanced Materials (CIMAV), Chihuahua 31136, Mexico

**Keywords:** oral dysbiosis, neuroinflammation, oral-brain axis, oral–gut–brain axis, periodontal pathogens, Alzheimer’s disease, Parkinson’s disease, depression, anxiety, microbiome modulation

## Abstract

Background: Growing evidence indicates that oral microbiome dysbiosis contributes to systemic inflammation, immune activation, and neural dysfunction. These processes may influence the onset and progression of major neuropsychiatric and neurodegenerative disorders. This review integrates clinical, epidemiological, and mechanistic findings linking periodontal pathogens and oral microbial imbalance to Alzheimer’s disease (AD), Parkinson’s disease (PD), depression, and anxiety. Methods: A narrative review was conducted using PubMed/MEDLINE, Scopus, Web of Science, and Google Scholar to identify recent studies examining alterations in the oral microbiota, microbial translocation, systemic inflammatory responses, blood–brain barrier disruption, cytokine signaling, and neural pathways implicated in brain disorders. Results: Evidence from human and experimental models demonstrates that oral pathogens, particularly *Porphyromonas gingivalis*, *Fusobacterium nucleatum*, and *Treponema denticola*, can disseminate systemically, alter immune tone, and affect neural tissues. Their virulence factors promote microglial activation, cytokine release (IL-1β, IL-6, TNF-α), amyloid-β aggregation, and α-synuclein misfolding. Epidemiological studies show associations between oral dysbiosis and cognitive impairment, motor symptoms in PD, and alterations in mood-related taxa linked to stress hormone profiles. Immunometabolic pathways, HPA-axis activation, and the oral–gut–brain axis further integrate these findings into a shared neuroinflammatory framework. Conclusions: Oral dysbiosis emerges as a modifiable contributor to neuroinflammation and brain health. Periodontal therapy, probiotics, prebiotics, synbiotics, and targeted inhibitors of bacterial virulence factors represent promising strategies to reduce systemic and neural inflammation. Longitudinal human studies and standardized microbiome methodologies are still needed to clarify causality and evaluate whether restoring oral microbial balance can modify the course of neuropsychiatric and neurodegenerative disorders.

## 1. Introduction

The relationship between the oral microbiome and the central nervous system, referred to as the oral–brain axis, has gained prominence as an essential contributor to the development and progression of neuropsychiatric disorders. Growing evidence indicates that oral microbial dysbiosis can initiate systemic inflammation, shape immune responses, and influence neurological signaling, thereby expanding our understanding of conditions once attributed predominantly to genetic or classical neurological factors [1,2]. Current research further highlights a strong association between shifts in microbial composition, particularly within the oral and gut microbiomes, and the onset of neuropsychiatric diseases [2]. The migration of oral bacteria into the gut may foster chronic inflammation, which, in turn, affects brain function and neurological health [3].

This review examines the multifaceted role of oral microbial dysbiosis in neuropsychiatric conditions, emphasizing the mechanisms that underpin these interactions and exploring therapeutic opportunities informed by this emerging evidence. Specifically, the direct and indirect pathways through which the oral microbiota influence brain function, including neuroinflammation, neurotransmitter regulation, and alterations to blood–brain barrier (BBB) integrity [3,4]. Particular attention is given to periodontal pathogens and their virulence factors, which have been implicated in neuroinflammation and neurodegeneration [5], as well as to the broader consequences of oral dysbiosis in disorders such as Alzheimer’s disease (AD), Parkinson’s disease (PD), anxiety, and depression [3,6]. We also consider the bidirectional communication within the oral–gut–brain axis, recognizing that inflammatory processes originating in the oral cavity may extend through the gastrointestinal tract and intensify neurological dysfunction [3]. Searching strategies are stated in Table 1.

Dysbiosis in the gut microbiota, characterized by altered microbial composition, diversity, and metabolic activity, has increasingly been recognized as a significant factor in the pathophysiology of neuropsychiatric disorders [7]. This imbalance can affect brain function through microbial metabolites, immune modulation, and neuroinflammatory pathways, influencing conditions such as autism spectrum disorder, anxiety, depression, and neurodegeneration [8].

Although the oral microbiome occupies a distinct niche, it also exerts notable effects on neurological health, either directly through neuronal pathways, such as the trigeminal and olfactory systems, or indirectly through systemic inflammatory pathways linked to the oral–gut–brain axis [3]. Chronic oral diseases can compromise the mucosal barrier, allowing microorganisms and endotoxins to enter the circulation, triggering systemic inflammation, and potentially disrupting the BBB [3]. Given the established influence of the gut microbiome on central nervous system function [8], we highlight the broader integration of oral health within the gut–brain framework, particularly in brain disorders [9]. This perspective underscores the need to consider oral health as a fundamental component in understanding and managing complex neuropsychiatric diseases. Building on this framework, it becomes essential to examine the composition and functional roles of the human oral microbiome as a distinct yet interconnected microbial ecosystem.

## 2. Human Oral Microbiome

The human oral microbiome (HOM) plays a fundamental role in overall health, contributing to functions such as protection against external pathogens, maintenance of immune homeostasis, and prevention of oral diseases, among others [10]. The HOM is considered the second largest microbial ecosystem in the human body, preceded only by the gut [11]. It is highly diverse and complex, comprising bacteria, fungi, archaea, and protozoa. Its composition varies depending on the different niches within the oral cavity (e.g., supragingival, subgingival, tongue, mucosal surfaces, saliva) and individual factors (e.g., age and development, hygiene habits, diet, and/or harmful habits) [12]. In addition to taxonomic diversity, recent evidence emphasizes that the functional output of the human microbiome is heavily influenced by microbiota-derived bioactive peptides [13]. These peptides include bacteriocins, microcins, lantipeptides, and other ribosomally synthesized and post-translationally modified peptides [13]. They play crucial roles in microbial competition, biofilm formation, immune modulation, and host–microbe communication. This functional, peptide-mediated layer offers a vital framework for understanding how microbial communities, including the oral microbiome, can impact local and systemic biological processes beyond mere microbial composition [13].

It has been shown that the oral microbiome dynamically interacts with the host via the oral–gut–brain axis. That dysbiosis promotes neuroinflammation and brain disorders [1,2,3]. Archaea and viruses modulate bacterial diversity and immunity [14,15]. The overlooked virome contributes through brain entry and alterations in microbiota-brain interactions [2,16].

### 2.1. Prokaryotes

#### 2.1.1. Bacteria

The oral cavity is estimated to harbor approximately 1000 bacterial species, primarily belonging to the phyla Firmicutes, Actinobacteria, Proteobacteria, Fusobacteria, Bacteroidetes, and Spirochaetes, which together account for 96% of the total oral bacteria [11]. Within these phyla, specific dominant genera can be identified. For instance, in Firmicutes, *Streptococcus*, *Veillonella*, and *Lactobacillus* prevail, playing essential roles in biofilm formation and the homeostasis of the oral ecosystem. In Actinobacteria, genera such as *Actinomyces* and *Corynebacterium* are abundant and crucial for colonizing dental surfaces and forming biofilms. Meanwhile, *Neisseria* and *Haemophilus* (Proteobacteria) are common in saliva and contribute to microbial balance. The species *Fusobacterium nucleatum* (Fusobacteria) is known for its role in microbial community cohesion within biofilms. In Bacteroidetes, significant genera include *Prevotella* and *Porphyromonas*, which interact with the oral immune system. Though less represented, Spirochaetes (e.g., *Treponema denticola*) are present and may influence biofilm dynamics [12,17,18,19,20].

The HOM is highly dynamic and varies due to several factors, including ontogeny. It has been observed that the microbiota is established during prenatal, perinatal, and postnatal phases, influenced by maternal transmission and environmental exposure [21,22]. Closely linked to development is diet, a key factor shaping the microbiome (including the oral, gut, and other microbiomes). Specifically, in HOM, high sugar and lipid intake promote the proliferation of *Streptococcus mutans*, *Lactobacillus*, *Neisseria*, and *Fusobacterium*, respectively [23,24]. Moreover, hygiene habits (e.g., mouthwash and antibiotic use) and behaviors such as smoking promote the enrichment of *F. nucleatum*, *Acinetobacter johnsonii*, *A. baumannii*, and *S. mutans*. In contrast, alcoholism favors the establishment and enrichment of *Actinomyces*, *Leptotrichia*, *Sphaerochaeta*, and members of the phylum Cyanobacteria [25,26].

In individuals without periodontal disease or advanced caries, the oral microbiota is characterized by the presence of *S. sanguinis*, *S. mitis*, *Neisseria* spp., *Haemophilus* spp., *Rothia* spp., *Veillonella* spp., and *Actinomyces*. These microorganisms maintain a dynamic balance with the host, preventing the proliferation of oral pathogens and supporting a symbiotic relationship [27]. However, specific bacterial phylotypes, such as *Porphyromonas gingivalis* (*P. gingivalis*), are implicated in periodontitis and the destruction of dental supporting tissues. *P. gingivalis* is known to disrupt host immune responses, evade defense mechanisms, and invade gingival epithelial cells, contributing to tissue damage [28]. Furthermore, *P. gingivalis* has been detected in the brains of Alzheimer’s disease patients, suggesting a possible link between periodontal and neurodegenerative diseases [29]. A significant proportion of bacterial-induced damage stems from metabolic activities, such as fermentation (e.g., lactic acid production) and proteolysis, among others [20].

#### 2.1.2. Archaea

Methanogenic archaea, particularly *Methanobrevibacter oralis*, may play a key role in biofilm formation and periodontal disease, with increased prevalence as the disease progresses. Their ability to consume hydrogen allows them to influence the balance of the microbial ecosystem, and they have also been identified in endodontic infections [30,31]. One major limitation in understanding their role in the oral cavity is that many aspects of their biology, such as cell wall composition and metabolism, remain unexplored. A significant limitation in comprehending the role of archaea within the oral microbiome stems from the fact that many fundamental aspects of their biology, including their distinctive cell wall composition and specific metabolic pathways, remain largely unexplored [32,33,34]. Methodological challenges exacerbate this knowledge gap: archaea are often overlooked in microbiome research, and many oral archaeal species are difficult to cultivate in laboratory settings [35,36]. Consequently, detailed metabolic and transcriptional studies, particularly at the community level, are scarce, limiting our ability to fully understand how these unique microorganisms influence oral health and disease [37]. Additionally, Dame-Teixeira et al. identified Thaumarchaeota and Methanocellales phylotypes in carious lesions and healthy biofilms, albeit in low concentrations [38]. The detection of archaea in carious biofilms suggests a potential influence on oral microbial ecology. These findings highlight the need for further research to elucidate their function in oral health and disease, and to integrate their taxonomy and metabolism into oral microbiome studies [38].

### 2.2. Eukaryotes

#### 2.2.1. Fungi

Although bacteria are the most diverse microorganisms in the HOM, fungi also form a crucial component of the oral microbiome. Recent studies have identified over 100 distinct fungal species residing in the oral cavity. Among them, *Candida* species, particularly *Candida albicans*, are the most prevalent [39,40]. These fungi typically exist as commensals but can become opportunistic pathogens under certain conditions, leading to infections such as oral candidiasis. Other commonly detected genera include *Cladosporium*, *Aureobasidium*, *Saccharomycetales*, *Aspergillus*, *Fusarium*, and *Cryptococcus*. Although these fungi are frequently present in the oral cavity, their roles in health and disease remain poorly understood [41].

*C. albicans* is the primary causative agent of oral candidiasis, particularly in immunocompromised individuals, where it proliferates on mucosal surfaces, forming biofilms and inducing infections. In dental caries, *C. albicans* and *C. dubliniensis* are found in carious lesions, interacting with *S. mutans* to enhance biofilm formation and acid production, which demineralizes enamel and worsens the disease. In periodontitis, *Candida* has been detected in periodontal pockets, potentially facilitating bacterial biofilm maturation and microbial colonization, although its impact remains to be thoroughly investigated [39,42]. On the other hand, Malassezia is an emerging component of the oral mycobiome, identified in healthy individuals through ITS sequencing. Unlike *Candida*, its role in the oral cavity is poorly understood, although it is thought to contribute to microbial balance. It has not been directly linked to caries, periodontitis, or oral candidiasis, but its interactions with other microorganisms remain uncertain. In studies on head and neck squamous cell carcinoma, Malassezia was more prevalent in patients with a better prognosis, suggesting a potential protective role. However, further research is needed to clarify its function in oral health and disease [42].

#### 2.2.2. Protozoa

Among the most studied protozoa in the oral cavity are *Entamoeba gingivalis* and *Trichomonas tenax* [43]. *E. gingivalis* is an anaerobic protozoan that inhabits the gums, dental plaque, and periodontal pockets. It can be transmitted through direct contact, such as kissing, or indirectly via contaminated food and utensils. Its prevalence is higher in individuals with periodontal disease: 88.9% in periodontitis, 84.9% in gingivitis, and 47.9% in healthy individuals. It contributes to inflammation and tissue destruction by interacting with host cells and promoting the production of pro-inflammatory molecules [44]. On the other hand, *T. tenax* is a flagellated protozoan that is commonly found in the oral cavity, particularly in individuals with poor oral hygiene and periodontal disease. It inhabits tartar, periodontal pockets, and tonsillar crypts. It is transmitted through saliva, aerosols, or contaminated utensils. Its prevalence is higher in individuals with periodontitis (25.6%) and gingivitis (5.7%) than in healthy individuals (3.2%). It can degrade periodontal tissues and induce inflammation [43,45].

### 2.3. Eukaryotic Viruses

The human herpes virus and human papillomavirus families are responsible for the most common primary viral infections of the oral cavity [46]. Eight distinct human herpes virus types have been identified, each causing unique primary or recurrent oral infections [47]. These include herpes simplex viruses 1 and 2, varicella-zoster virus, Epstein–Barr virus, and cytomegalovirus, all of which are double-stranded DNA viruses [48]. These viruses possess a conserved structural organization: a linear double-stranded DNA genome encased within an icosahedral capsid, enveloped by tegument proteins and a host-derived lipid bilayer [49,50]. Epstein–Barr virus and cytomegalovirus, known for their associations with marginal periodontitis, are also implicated in the inflammatory processes leading to periapical bone destruction [46]. Further investigations link herpesviral lytic proteins to the activation of cellular signaling pathways, such as Notch signaling, and to increased expression of pro-inflammatory cytokines [46]. This biological response promotes the transcription of receptor activator of nuclear factor kappa-B ligand, which subsequently drives osteoclastogenesis and bone resorption [46]. The heightened production of pro-inflammatory cytokines further intensifies bone resorption in the periapical region [51].

### 2.4. Prokaryotic Viruses

Prokaryotic viruses, commonly referred to as bacteriophages, are the most prevalent biological entities identified to date [46,52]. Bacteriophages, which are viruses that infect bacteria, can either persist within host cells as parasitic deoxyribonucleic acid or harness bacterial metabolic machinery for their replication, culminating in the lysis of the host bacterium [53].

Notably, bacteriophages constitute a larger proportion of the human virome than their eukaryotic counterparts, despite their limited representation in current genomic databases [46]. Similarly to eukaryotic viruses, the taxonomic classification of bacteriophages is determined by various attributes, including the molecular composition of their viral genome, the structure of their capsid, the presence of a viral envelope, their host range, and shared genomic sequences [46]. Bacteriophages exhibit diverse morphologies and genomic compositions [53,54]. They can possess a proteinaceous capsid, which may be tailed and enclose a double-stranded deoxyribonucleic acid genome, or be non-tailed and accommodate either double-stranded DNA, single-stranded DNA, or ribonucleic acid genomes. Filamentous and pleomorphic phage types are also observed. Tailed bacteriophages constitute the majority (96%) of isolates and are classified into families based on their tail characteristics. For instance, large virions featuring a long contractile tail are classified under the families *Myoviridae*, *Ackermannviridae*, or *Herelleviridae*. Those with a long, flexible, but non-contractile tail belongs to the *Siphoviridae* family, while small virions with a short, non-contractile tail are assigned to the *Podoviridae* family. Phages with huge genomes are further grouped as jumbo phages and megaphages [53].

## 3. Linking Microbiome Dysbiosis in the Oral–Brain Axis to Neuroinflammation

The prevalence of neuropsychiatric disorders has increased in recent years, particularly following the COVID-19 pandemic [15]. Major neuropsychiatric disorders include AD, PD, depression, and anxiety [15]. These conditions can significantly affect individuals and their families, posing a substantial global public health challenge [15]. Research suggests that the etiology of these diseases involves both genetic and environmental factors; however, the host’s microbiota has also been identified as a critical factor in the development of neuropsychiatric disorders [2,15].

It has been established that the brain exhibits greater interconnectedness with systemic health than previously understood [2]. A significant bidirectional relationship exists between the intestinal microbiota and the brain, commonly referred to as the gut–brain axis [55,56,57]. The intricate gut–brain communication network involves the enteric nervous system, the sympathetic and parasympathetic branches of the autonomic nervous system, and neuroendocrine and neuroimmune signaling pathways. Visceral information travels from the intestines to the spinal cord and the nucleus of the solitary tract via afferent spinal and vagal sensory nerves, influencing higher brain regions such as the hypothalamus and limbic forebrain via polysynaptic pathways [58]. Likewise, the human oral microbiome may affect brain function through both direct and indirect routes, including neural pathways such as the trigeminal and olfactory systems, as well as the broader oral–gut–brain axis [3]. Although these connections are increasingly acknowledged, the specific biological mechanisms and pathways linking oral microbial communities to neuropsychiatric conditions remain insufficiently characterized [2]. In contrast to the extensive research devoted to the gut–brain axis in the context of mental health, the contribution of the oral microbiome to psychiatric disorders has received comparatively limited investigation [3,59,60,61]. Individuals with psychiatric disorders frequently display suboptimal oral hygiene and impaired periodontal health [62]. A meta-analysis demonstrated that these disorders are associated with an increased risk of dental caries, as evidenced by elevated decayed, missing, and filled teeth indices and increased tooth loss [15,63]. Furthermore, another meta-analysis indicated a 50-fold increased risk of periodontal disease among patients with severe mental illness (95%) [62,64].

**Figure 1:**
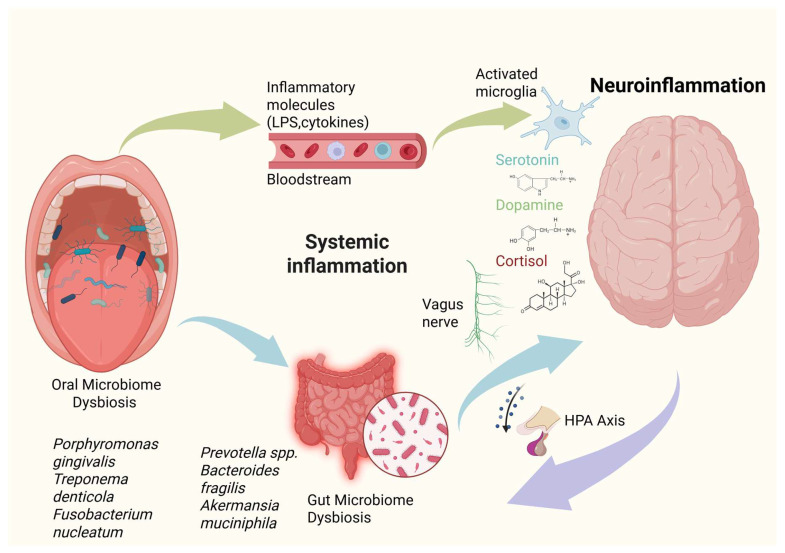
Schematic representation of the oral–gut–brain axis linking microbiome dysbiosis to neuroinflammation. Oral microbiome dysbiosis, characterized by pathogens such as *Porphyromonas gingivalis*, *Treponema denticola*, and *Fusobacterium nucleatum*, promotes systemic dissemination of inflammatory mediators (e.g., lipopolysaccharides and pro-inflammatory cytokines). Through the oral–gut axis, these inflammatory signals and translocated microbial products influence gut barrier integrity and microbial composition, promoting gut microbiome dysbiosis. This gut imbalance, enriched in taxa such as *Prevotella* spp., *Bacteroides fragilis*, and *Akkermansia muciniphila*, further amplifies systemic inflammation and metabolic signaling. Immune mediators and microbial metabolites influence brain function via hematogenous routes, vagus nerve signaling, and hypothalamic–pituitary–adrenal (HPA) axis activation, leading to microglial activation, altered neurotransmitter balance (serotonin, dopamine), and increased cortisol, ultimately promoting neuroinflammation. This integrated framework highlights the bidirectional communication between microbial communities, systemic inflammation, and central nervous system health.

Given that the mouth is the main gateway into the human body, alterations in its microbial community may contribute to the onset and worsening of psychiatric and neurodegenerative conditions [15] (Figure 1).

Pathways involved in oral microbiome dysbiosis-mediated neuroinflammation and neurodegeneration include the systemic spread of bacterial components and metabolites, direct invasion of the central nervous system, and activation of immune responses that cross-react with neural antigens [65].

### 3.1. Immune Pathways

Elevated circulating pro-inflammatory cytokines, such as IL-1β and TNF-α, can cross the blood–brain barrier, exacerbating neurological deficits. These cytokines act as key signaling molecules in neuroinflammation, with TNF-α influencing central nervous system development, neuronal plasticity, cognition, and behavior [66]. In Alzheimer’s disease, periodontal inflammation induces IL-1β, IL-6, IL-8, TNF-α, and CRP, promoting brain inflammation [67]. Pathogens like *P. gingivalis* may transport amyloid peptides from peripheral sites to the liver, exacerbating systemic pathology [68].

Periodontitis indirectly affects anxiety and depression via cytokines that activate TNF-α- and IL-1 receptor-expressing endothelial cells [69]. This stimulates perivascular macrophages and microglia, inducing neuroinflammation [69]. Compromised periodontal integrity also allows lipopolysaccharides to enter circulation, activating the hypothalamic–pituitary–adrenal axis and elevating stress hormones.

In PD, elevated gingival crevicular fluid IL-1β signals local inflammation, and poor oral health worsens progression [70]. α-synuclein (ASN) levels in saliva correlate with disease duration and severity, serving as a non-invasive biomarker. Periodontitis-driven systemic inflammation disrupts blood–brain barrier integrity, activates microglia, and promotes immune cell brain infiltration [67,71,72,73,74].

### 3.2. Metabolic Pathways

The translocation of oral bacteria and their metabolic products into the circulatory system and central nervous system can adversely impact brain function by triggering a neuroinflammatory cascade. This response involves the upregulation of inflammatory signaling molecules, including pro-inflammatory cytokines, which can lead to a persistent, albeit potentially low-grade, immune reaction. Such systemic inflammation can compromise neurovascular integrity, leading to increased BBB permeability, diminished nutrient supply, and accumulation of toxic substances within the brain [2].

### 3.3. Neural Pathways

The aberrant localization of microorganisms within the host can precipitate disease, particularly when oral microbial communities exhibit dysbiosis. During such states, an overabundance of specific pathogenic microbes can arise. Subsequently, these oral microorganisms and their byproducts may disseminate via the bloodstream to various tissues, such as the cardiovascular system, to reach the brain, where they are implicated in impairing neurological functions and causing neurodegeneration through the accumulation of toxic substances [2].

### 3.4. Direct Invasion: Oral Bacteria Crossing the Blood–Brain Barrier

The bacterium *P. gingivalis* has been observed to enter the bloodstream and migrate to the brain, where it establishes colonies and secretes neurotoxic enzymes known as gingipains [2]. These gingipains are implicated in the aberrant processing of the transmembrane protein amyloid precursor protein (APP), which plays a crucial role in maintaining synaptic stability, neuronal growth, and protection [2]. This disruption leads to the miscleavage of APP, resulting in the formation of amyloid beta protein plaques that subsequently trigger neuronal demise [2].

### 3.5. Overlapping Genomic and Molecular Activities in Neurodegenerative Diseases

Growing transcriptomic and proteomic evidence shows an overlap in the molecular activities of PD, AD, and other dementias. Common differentially expressed genes have been identified across these diseases, linked to processes such as chemical synaptic transmission and nervous system development [75,76,77,78,79,80,81,82]. At the protein level, studies show similar dysregulation of proteins in AD brains and Tau pathologies, implicating new proteins in the disease process [83,84]. These similarities suggest a deep molecular convergence, even if the affected brain regions may vary clinically [79,85,86].

## 4. Oral–Gut–Brain Inflammatory Axis

The oral–gut–brain axis allows bidirectional microbial transfer, with oral bacteria traveling to the gut via saliva to promote systemic inflammation and influence brain health [3,87]. Oral dysbiosis, as seen in periodontitis, damages mucosal barriers, allowing pathogens and lipopolysaccharides (LPS) endotoxins to enter the bloodstream, cause neuroinflammation, and weaken the BBB [3,88].

### 4.1. Bacterial Pathogen Evidence: Diversity, Richness, Abundance in Fecal vs. Oral Microbiomes

Fecal and oral microbiomes exhibit site-specific differences, with shared amplicon sequence variants primarily originating from the oral cavity, indicating primary oral-to-gut transmission [3]. In PD, alpha-diversity increases in stool and saliva compared to controls, characterized by an overabundance of *Streptococcus mutans* and *Bifidobacterium dentium* [89,90]. Beta-diversity significantly differs in PD gut and oral samples from controls, with gut depletion of short-chain fatty acid producers (*Prevotella*, *Roseburia*) and enrichment of Escherichia and *Lactobacillus* [91]. In Alzheimer’s disease, oral microbiomes show increased levels of Firmicutes and Fusobacteria with greater cognitive decline, while Proteobacteria decrease; conversely, the gut microbiome shows a declining Firmicutes/Bacteroidetes ratio [92]. PD salivary and plaque microbiotas cluster separately from controls, often without changes in alpha-diversity [93]. Patterns of oral dysbiosis in neurodegenerative diseases mirror gut changes observed in AD and PD [3].

### 4.2. Virulence Factors from Oral and Gut Pathogens Supporting Axis Communication Porphyromonas gingivalis

*P. gingivalis* enters circulation, colonizes the brain, and secretes gingipains that disrupt BBB and promote Aβ/tau pathology [1,94]. Gut dysbiosis worsens this via leaky gut, allowing microbial products to reach the brain through vagal/enteric pathways [95,96]. Gingipains are found in >90% of AD brains, correlating with tau tangles and cognitive decline [97,98]. They degrade proteins, evade immunity, and cleave APP to increase Aβ1–42 [1,3]. Secreted in vesicles, gingipains breach the BBB by degrading tight junctions, upregulating transcytosis, and activating inflammasomes, leading to Aβ plaques and neurodegeneration [1,99]. In PD, gingipains are found in the substantia nigra [100].

## 5. Alzheimer’s Disease

AD is a neurodegenerative disorder resulting from the interplay of environmental, genetic, and epigenetic factors [101]. As the leading cause of dementia, it has rapidly become one of the most costly and lethal diseases of the 21st century, and is currently the seventh leading cause of death worldwide [102,103]. The initial symptoms typically include memory loss, cognitive dysfunction, and language disorders. As the disease advances, disorientation and behavioral disorders manifest. In the final stages, patients become unable to speak, walk, or even eat [103,104]. In 2021, the prevalence of this disease was estimated at more than 50 million people worldwide, and this number is expected to triple by 2050 [105]. Approximately two-thirds of individuals with AD are women, and one-third are men, a disparity attributed to genetic factors and women’s longer life expectancy. Hispanics and African Americans have a 1.5 times greater likelihood of developing AD.

### 5.1. Pathophysiology of Alzheimer’s Disease

The overproduction of amyloid-beta, a normally soluble protein, is believed to trigger its self-assembly into oligomers and subsequently into highly ordered amyloid fibrils, which manifest as plaques observable in pathological specimens [106].

Evidence indicates that both soluble Aβ and amyloid plaques exert toxic effects. These impacts on cellular functions are thought to initiate secondary or downstream processes, including tau protein hyperphosphorylation, inflammation, oxidative stress, and excitotoxicity. Consequently, these events result in cell death and deficits in neurotransmitters, particularly acetylcholine, whose reduction contributes to certain symptoms of Alzheimer’s disease [106].

### 5.2. The Link Between Oral Microbiome, Periodontal Diseases and Alzheimer’s Disease

**Figure 2:**
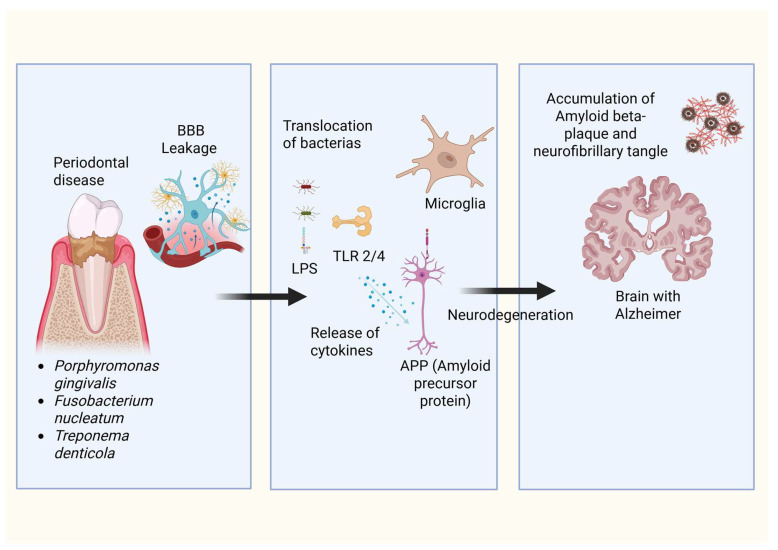
Proposed mechanism linking periodontal disease–associated bacteria to Alzheimer’s disease pathology. Specific periodontal pathogens, including *Porphyromonas gingivalis*, *Fusobacterium nucleatum*, and *Treponema denticola*, are implicated in the compromise of the blood–brain barrier integrity, thereby enabling the transmigration of bacterial constituents and pro-inflammatory mediators into cerebral parenchyma. Such invasive elements subsequently trigger microglial activation via Toll-like receptors. This activation elicits the release of cytokines and modulates the processing of amyloid precursor protein. The cumulative effects of this neuroinflammation, coupled with aberrant amyloid-beta and tau protein accumulation, precipitate the neurodegenerative processes emblematic of Alzheimer’s disease pathology.

*P. gingivalis* can traverse the oral mucosal barrier to enter the bloodstream, potentially breaching the BBB and inducing the accumulation of beta-amyloid plaques and neurofibrillary tangles following experimental oral infection in mice [29,107]. Furthermore, *P. gingivalis* lipopolysaccharide has been detected in human brains affected by AD suggesting that *P. gingivalis* brain infection may be an essential factor in the development of AD [29]. Similarly, *P. gingivalis* DNA has been identified in Alzheimer’s brains, as well as in the cerebrospinal fluid of living patients diagnosed with AD, suggesting its potential utility as a diagnostic marker [29]. Other bacterial species, such as *Actinomycetes*, *Helicobacter pylori*, and *Chlamydia pneumoniae*, as well as certain fungi and Herpes Simplex virus type 1 (HSV-1), have also been detected in post-mortem examinations of individuals with AD [108]. Perivascular spaces may enable bacteria and their products to access the brain [68] directly. Additionally, circumventricular organs might facilitate the entry of substances that typically cannot cross the BBB. Bacterial species can also reach the central nervous system (CNS) via peripheral nerves, following their pathways [108]. However, the presence of these bacteria in peripheral nerve fibers or in systemic circulation does not automatically guarantee their access to the CNS [67]. It is hypothesized that various cofactors, such as pro-inflammatory cytokines, concurrent infections, and the patient’s age, may contribute to the entry of periodontal bacteria into the CNS [67] (Figure 2).

### 5.3. Oral Biologic Responses and Molecular Mechanisms for Alzheimer’s Disease

Oral pathogens such as *P. gingivalis* can translocate to AD brains, inducing amyloid-beta production and tau hyperphosphorylation [109,110]. Its LPS worsens amyloid-beta aggregation and contributes to AD-like pathology [98,111], while oral dysbiosis in AD patients triggers immune responses that sustain inflammation [3].

**Figure 3:**
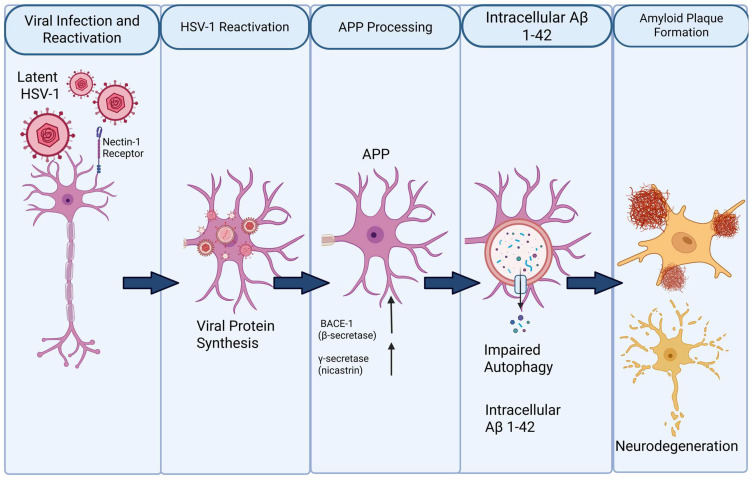
Proposed mechanism linking HSV-1 reactivation to amyloid-β (Aβ) pathology and neurodegeneration. Latent herpes simplex virus type 1 (HSV-1) persists in neurons and reactivates upon stress or immunological triggers through interaction with the nectin-1 receptor. Viral reactivation induces intracellular viral protein synthesis and alters host cellular pathways, including amyloid precursor protein (APP) processing. Up-regulation of β-secretase (BACE-1) and γ-secretase favors amyloidogenic cleavage of APP, leading to increased generation of Aβ, particularly the Aβ1–42 species. Concurrent impairment of autophagy reduces intracellular clearance, promoting accumulation of Aβ1–42 within neurons. Progressive intracellular Aβ burden contributes to extracellular amyloid plaque formation, synaptic dysfunction, and ultimately neurodegeneration.

HSV-1, a neurotropic virus commonly detected in the oral cavity and trigeminal ganglion, has been increasingly recognized as a modulator of amyloidogenic pathways implicated in AD [112,113,114]. Upon neuronal entry via receptors such as nectin-1, HSV-1 is trafficked into early and late endosomal compartments, which coincide with the principal intracellular sites of APP cleavage [112,113,114,115]. Experimental evidence demonstrates that HSV-1 infection enhances the expression and enzymatic activity of β-site APP-cleaving enzyme 1 (BACE-1) and the γ-secretase complex, thereby favoring amyloidogenic APP processing and leading to intracellular accumulation of amyloid-β (Aβ), particularly the aggregation-prone Aβ1–42 isoform [112,113,114]. Importantly, Aβ accumulation is initially observed within neurons rather than extracellularly, indicating that HSV-1 promotes early intracellular amyloid pathology preceding plaque deposition [112,113]. Accumulating evidence indicates that intraneuronal Aβ exerts pronounced neurotoxic effects and represents a critical early event in AD pathogenesis, preceding extracellular plaque formation and neuronal loss [113,116] (Figure 3).

A key mechanism by which the periodontal pathogen *P. gingivalis* perturbs host cell function involves the release and cellular uptake of outer membrane vesicles (OMVs). These vesicles, which encapsulate virulence determinants such as LPS and gingipains, readily interact with epithelial and immune cells. Experimental evidence indicates that OMVs derived from *P. gingivalis* enter host cells through a lipid raft–dependent endocytic process that is independent of clathrin and caveolin but relies on Rac1-controlled micropinocytosis [117]. Once internalized, OMVs are transported through the endosomal system, initially localizing to EEA1-positive early endosomes and subsequently accumulating within LAMP1-positive late endosomes and lysosomes [117,118]. Notably, OMVs are not rapidly degraded within these compartments; instead, they persist intracellularly while retaining enzymatic activity of associated gingipains. Their prolonged residence within acidified vesicles suggests continuous disruption of endosomal–lysosomal integrity, a cellular network essential for proteostasis, autophagic flux, and controlled protein degradation [118,119].

Chronic systemic exposure to *P. gingivalis* LPS in murine models results in neuroinflammatory responses accompanied by intracellular accumulation of amyloid-β (Aβ) [120]. Importantly, this phenotype is dependent on cathepsin B activity, indicating that lysosomal protease dysregulation links inflammatory signaling to abnormal amyloid processing [120,121]. In addition to disrupting vesicular trafficking, *P. gingivalis* exerts strong effects on host redox signaling by inducing nitrosative stress. Infection with *P. gingivalis* or stimulation with its LPS markedly increases expression of inducible nitric oxide synthase (iNOS) in multiple cell types, including endothelial cells and macrophages [122].

**Figure 4:**
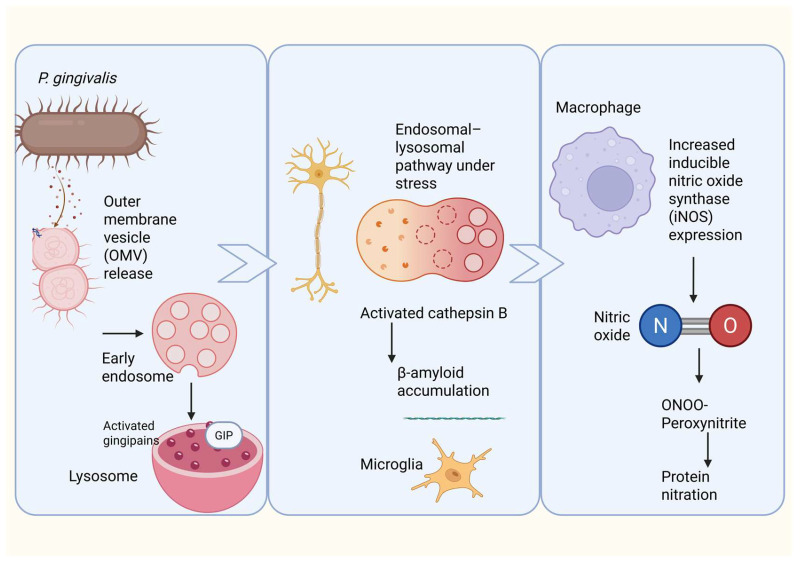
Proposed mechanism linking *Porphyromonas gingivalis* outer membrane vesicles to neuroinflammation, β-amyloid accumulation, and nitrosative stress. *Porphyromonas gingivalis* releases outer membrane vesicles (OMVs) containing virulence factors, including gingipains, which are internalized by neural cells through the endosomal–lysosomal pathway. Under cellular stress, lysosomal dysfunction leads to activation and leakage of cathepsin B, promoting intracellular β-amyloid accumulation and microglial activation. In parallel, OMV-mediated stimulation of macrophages and microglia induces upregulation of inducible nitric oxide synthase (iNOS), leading to excessive nitric oxide (NO) production. NO reacts with superoxide to form peroxynitrite (ONOO^−^), driving protein nitration and amplifying neuroinflammation and neurodegenerative processes associated with Alzheimer’s disease.

Macrophage-based models further demonstrate that *P. gingivalis* LPS induces sustained iNOS expression and prolonged NO release via Toll-like receptor–mediated signaling pathways [123]. Under inflammatory conditions, elevated NO readily reacts with superoxide radicals to form peroxynitrite (ONOO^−^), a highly reactive nitrogen species capable of inducing protein nitration, mitochondrial damage, and lipid oxidation. Peroxynitrite-driven post-translational modification of neuronal proteins has been strongly implicated in neurodegenerative cascades, providing a mechanistic link between chronic periodontal inflammation, nitrosative stress, and neuronal vulnerability [124] (Figure 4).

### 5.4. Metabolic Biochemistry Connecting Oral Bacteria to Alzheimer’s Disease Symptoms

The emerging field of metabolic biochemistry links oral bacteria to AD, focusing on microbial metabolites that influence neuroinflammation and neurotransmission [37]. Oral bacteria produce metabolites that modulate inflammation, immune responses, and neurotransmitters, including tryptophan metabolism, which alters serotonin levels implicated in AD [125]. Some bacteria generate short-chain fatty acids with anti-inflammatory effects, though dysbiosis may promote systemic inflammation [126]. Oral pathogens also trigger vascular inflammation and oxidative stress, contributing to neurodegeneration [3].

## 6. Anxiety and Depression

Depression and anxiety disorders are highly prevalent in both the general population and primary care settings [127]. It Is common for individuals with depression to exhibit symptoms of anxiety disorders, and vice versa. These conditions can coexist, with individuals meeting the diagnostic criteria for both [127]. While differentiating between them can be challenging, it is crucial to diagnose and treat both conditions due to their significant impact on health and survival [127]. Mental illnesses significantly impact patients’ academic pursuits, professional lives, and daily functioning, with severe cases potentially leading to suicidal behavior [128]. These conditions also affect the well-being of patients’ families. Globally, mental disorders have become a significant public health issue, imposing substantial medical costs and economic losses [128].

Depression is a complex mental health disorder that, in 2017, was the leading cause of disability worldwide, affecting over 322 million individuals, while just over 260 million people suffered from anxiety disorders [69,129]. Both conditions are more prevalent in women, with an approximate 2:1 ratio compared with men, and exhibit a comorbidity phenomenon [130]. It is estimated that approximately 45.7% of individuals with depression have a history of one or more episodes of anxiety [130].

### 6.1. Pathophysiology of Anxiety and Depression

Genetic factors are considered to play an essential role in the predisposition to these diseases; however, non-genetic risk factors such as trauma, poor upbringing or lifestyles, and current exposure to stress are also associated with the development of depression and anxiety [130]. Modern living, with its associated high stress, reliance on processed foods, overemphasis on hygiene, and prevalent antibiotic use, coupled with environmental shifts such as elevated pollution, climate instability, and urban expansion, has contributed to a transformation of the human microbiota into an industrialized pattern [69]. These modifications in microbial composition, in conjunction with a decline in particular functional capabilities, could foster microbial communities that are less effective at preventing disease and more prone to promoting it, thereby exacerbating existing mental health issues [69].

### 6.2. The Link Between Oral Microbiome and Anxiety and Depression

Recent studies have linked periodontal pathogens to the etiology and pathophysiology of depression and anxiety disorders [131]. In a survey of self-reported anxiety and depressive symptoms among adolescents, no significant differences were observed in oral microbiome diversity between the anxiety or depression groups and controls. Nonetheless, these symptoms correlated with altered relative abundances of specific taxa, including a positive association of Spirochaetaceae with both anxiety and depression, as well as positive correlations of *Actinomyces*, *Fusobacterium*, and *Leptotrichia* spp. with depression. Additionally, *Leptotrichia* spp. relative abundance positively correlated with cortisol levels [132,133]. Furthermore, in a non-clinical adult population, the overall composition of the oral microbiome, together with diurnal fluctuations in the relative abundance of bacterial taxa, varied with psychological distress and affective state [129].

Interestingly recent evidence suggests that specific alterations in the oral microbiome are associated with depressive symptoms during pregnancy [134]. In particular, studies examining pregnant cohorts have reported significant reductions in the relative abundance of *Neisseria*, *Fusobacterium*, *Capnocytophaga*, and *Streptococcus* in women exhibiting clinically relevant depressive symptoms [134]. Conversely, increased abundance of the phyla Firmicutes and Spirochaetes has been observed among participants with more severe depressive profiles [134]. These findings indicate that pregnancy-related depression may be linked not to overall microbial diversity but rather to shifts in key bacterial genera, which could reflect underlying immunological, metabolic, or inflammatory processes unique to the perinatal period [134].

**Figure 5:**
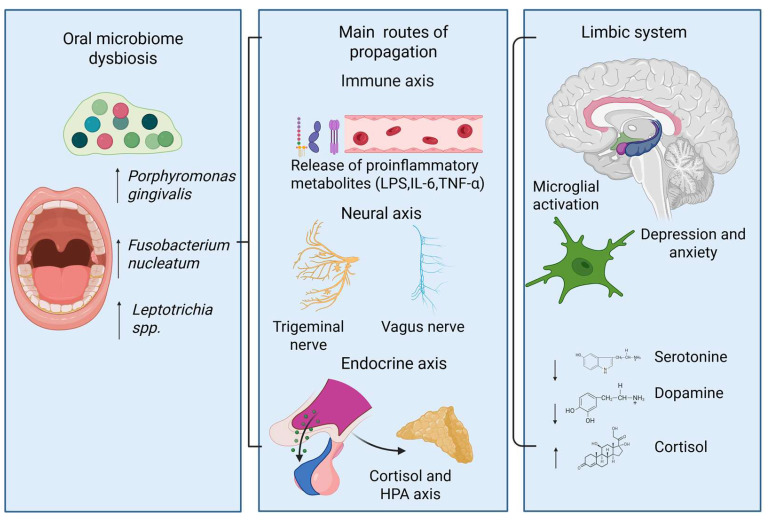
Proposed mechanisms connect dysbiosis in the oral microbiome and mood disorders. Specifically, an unhealthy microbial profile—characterized by increased levels of periodontal pathogens—can initiate a body-wide immune response and release inflammatory mediators. These inflammatory signals can reach the brain via immune, neural, and endocrine pathways, leading to activation of the hypothalamic–pituitary–adrenal axis and subsequent cortisol release. Within the brain, this can lead to microglial activation and alterations in neurotransmitters within limbic circuits, which are associated with symptoms of depression and anxiety.

Periodontal bacteria can directly access and affect the brain through multiple routes [69,131] (Figure 5).

### 6.3. Oral Biologic Responses and Molecular Mechanisms for Anxiety and Depression

The oral microbiome affects anxiety and depression through systemic inflammation, direct neural pathways such as the trigeminal nerve and the olfactory system, and the oral–gut–brain axis [59]. The oral microbiota also dysregulates the hypothalamic–pituitary–adrenal axis: stress alters the microbial composition and virulence of periodontitis-related species [2] while salivary cortisol levels associate with psychological symptoms and bacterial abundance [135]. Notably, specific changes include lower *Neisseria elongata* and higher *Oribacterium asaccharolyticum* in anxiety [3], increased *Prevotella nigrescens* and *Neisseria* spp., and decreased *Rothia* and *Streptococcus* in depression [129]. Genome-wide studies also reveal interactions between salivary-tongue microbiota and these disorders [3].

### 6.4. Metabolic Biochemistry Connecting the Oral Bacterial Component to Anxiety and Depression

The metabolic biochemistry linking oral bacteria to anxiety and depression involves neuroactive metabolites and modulation of host pathways. Oral bacteria produce or break down neurotransmitters like serotonin, dopamine, GABA, and others, helping maintain neurochemical balance even in small amounts [59]. Tryptophan metabolism, common in oral species, produces serotonin, a mood regulator, with lower plasma levels found in symptomatic groups [3,69,125]. Additionally, pathways involved in tyrosine metabolism, glutamate-glutamine, and glutamatergic synapses influence reward circuits linked to mood disorders [2].

## 7. Parkinson’s Disease

PD is a prevalent neurodegenerative disorder resulting from the death of dopaminergic neurons in the substantia nigra pars compacta [135]. It is characterized by both motor symptoms (MS), such as tremors, and non-motor symptoms (NMS), such as constipation [90,135].

PD leads to escalating motor impairments that diminish daily functioning and overall quality of life [136]. The primary motor symptoms, which are foundational to current diagnostic standards, manifest early [136]. However, the progression of motor disability is significantly influenced by the emergence of postural instability, worsening gait issues, and difficulties with swallowing (dysphagia) and speech (dysarthria) [136].

PD is the second most frequent neurodegenerative condition, affecting over 6 million people worldwide [136]. This represents a 2.5-fold rise in occurrence compared to the previous generation, establishing PD as a major contributor to neurological impairment [136]. Over the last 20 years, the occurrence and prevalence of this condition have increased significantly and unexplainedly [137]. Men are more likely to be affected than women, with a roughly 3-to-2 ratio [136].

### 7.1. Pathophysiology of Parkinson’s Disease

The etiology of PD remains unknown in the vast majority of cases, but is believed to involve a complex interaction between genetic predisposition, environmental factors, and age-related processes [95]. Furthermore, certain ecological and behavioral factors, potentially amenable to modification, have been identified as contributing to the development of PD across diverse populations [136]. A conclusive diagnosis of PD necessitates the post-mortem identification of characteristic neuropathological alterations within the brain [137]. Pathologically, the disease is characterized by the aggregation of ASN into Lewy bodies and Lewy neurites, a process associated with a dense cellular environment rich in membranes, vesicular structures, malformed organelles such as mitochondria, and a high lipid content [137]. Emerging research indicates that comparable pathological changes occur in peripheral organs, such as the skin, colon, and salivary glands, even at the earliest stages of the disease, suggesting that PD affects multiple bodily systems [137]. Aggregates of phosphorylated ASN, the hallmark histopathological feature of PD, have been found not only in the central nervous system but also within neurons of the olfactory bulb and in the peripheral autonomic nervous system of the upper aerodigestive and gastrointestinal tracts, at a very early stage of the disease, even before motor symptoms appear [95].

Inflammation is a critical component of PD [138]. In post-mortem examinations, microglial cells within the substantia nigra of individuals with PD exhibit heightened activation compared to those in healthy brains [70]. Elevated levels of inflammatory cytokines have been detected in the blood, cerebrospinal fluid, colon, and saliva of PD patients compared with healthy controls [70]. Microglial activation leads to the secretion of inflammatory mediators that can damage the central nervous system and contribute to neurodegeneration [138]. Evidence from multiple research works indicates that PD correlates with a dysbiosis in the host-microbiota relationship within both the oral cavity and the intestinal tract [70].

### 7.2. The Link Between Oral Microbiome and Parkinson’s Disease

**Figure 6:**
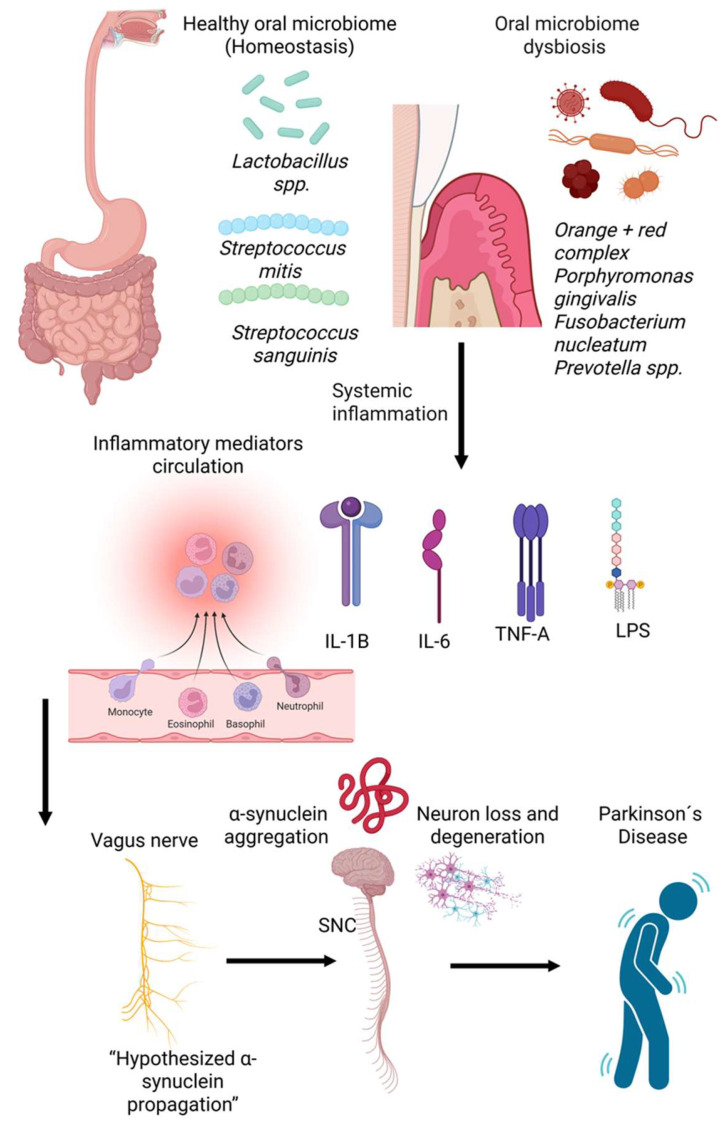
Conceptual model linking oral microbiome dysbiosis to Parkinson’s disease (PD) pathogenesis. A balanced oral microbiome dominated by commensals such as *Lactobacillus* spp., *Streptococcus mitis*, and *Streptococcus sanguinis* supports homeostasis. In contrast, periodontal dysbiosis is characterized by pathogens including *Porphyromonas gingivalis*, *Fusobacterium nucleatum*, and *Prevotella* spp. promotes systemic inflammation and circulating inflammatory mediators (e.g., IL-1β, IL-6, TNF-α, and LPS). These inflammatory signals and microbial components may influence neuroinflammatory pathways and facilitate α-synuclein misfolding and aggregation, potentially propagating along the vagus nerve to the substantia nigra, contributing to neuronal degeneration and PD development.

Recent research has established a connection between oral microbiota and PD, revealing distinct oral bacterial compositions in individuals with PD compared to healthy subjects [70,139,140]. Although the oral microbiota of early-stage PD patients was comparable to that of control groups, patients exhibited higher proportions of Firmicutes, Negativicutes, *Lactobacillaceae*, *Lactobacillus*, *Scardovia*, *Actinomyces*, *Veillonella*, *Streptococcus mutans*, and *Kingella oralis* [70]. Conversely, *Lachnospiraceae* and *Treponema* were found in lower quantities. The major periodontitis pathogens, including *Aggregatibacter actinomycetemcomitans*, *Porphyromonas gingivalis*, *Tannerella forsythia*, *Treponema denticola*, *Prevotella intermedia*, and *Fusobacterium nucleatum*, may induce neurodegeneration by directly invading the brain or by colonizing other organs, leading to systemic inflammation [138] (Figure 6).

### 7.3. Oral Biological Responses and Molecular Mechanisms in Parkinson’s Disease

ASN is highly susceptible to nitrosative post-translational modification due to its intrinsically disordered structure and exposed tyrosine residues. Nitrated forms of ASN have been consistently detected in dopaminergic neurons and Lewy body inclusions in PD and related synucleinopathies, supporting a pathogenic role for nitrosative stress in disease progression [141,142,143]. Experimental evidence indicates that these modifications are predominantly mediated by peroxynitrite rather than nitric oxide (NO) alone, leading to altered protein conformation and impaired clearance [144].

ASN nitrosation typically arises under conditions of sustained inflammation, where inducible nitric oxide synthase (iNOS) is upregulated, resulting in prolonged NO production [145]. Simultaneously, inflammatory oxidative stress increases superoxide generation. The rapid reaction between NO and superoxide produces peroxynitrite, a highly reactive nitrogen species that nitrates tyrosine residues within ASN [146]. Nitrated ASN exhibits increased resistance to proteasomal and lysosomal degradation and shows an enhanced tendency to form stable oligomeric and fibrillar assemblies, which are widely considered neurotoxic [143,147].

**Figure 7:**
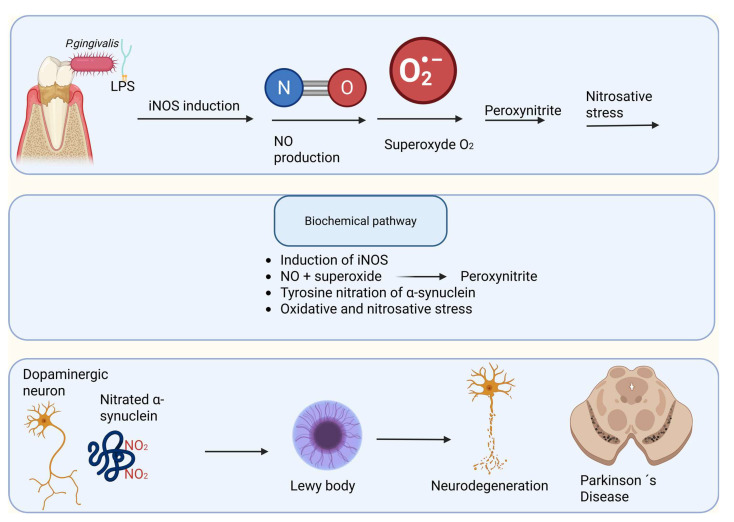
Nitrosative stress–mediated α-synuclein pathology linking *Porphyromonas gingivalis*–derived lipopolysaccharide to Parkinson’s disease. Lipopolysaccharide (LPS) from *Porphyromonas gingivalis*, a keystone periodontal pathogen, induces the expression of inducible nitric oxide synthase (iNOS) in peripheral and central immune cells, leading to excessive nitric oxide (NO) production. NO rapidly reacts with superoxide anion (O_2_•^−^) to generate peroxynitrite (ONOO^−^), a potent reactive nitrogen species that drives nitrosative stress. Peroxynitrite mediates tyrosine nitration of α-synuclein, promoting its misfolding, aggregation, and incorporation into Lewy bodies within dopaminergic neurons. Progressive accumulation of nitrated α-synuclein contributes to neuronal dysfunction, neurodegeneration, and the development of Parkinson’s disease.

Experimental studies have demonstrated that *P. gingivalis* and its LPS induce robust iNOS expression and sustained NO release in endothelial and immune cells [122]. When sustained over time, such a peripheral inflammatory environment may intersect with brain regions already vulnerable to oxidative stress, thereby promoting conditions favorable for ASN nitrosation and aggregation. Importantly, this mechanism does not require direct bacterial invasion of the CNS; instead, oral pathogens may act indirectly by amplifying systemic inflammatory and nitrosative pathways that drive pathological protein modification [148,149] (Figure 7).

Bacteriophages, abundant in the oral and gut virome, regulate prokaryotic composition through lysis and lysogeny, affecting permeability, inflammation, and metabolite production [150,151,152]. In PD, gut phagobiota shows altered diversity and abundance, for example, increased levels of *Siphoviridae* and *Myoviridae*, correlating with ASN pathology and the loss of dopaminergic neurons [152,153,154]. Phages may directly induce ASN misfolding or do so by influencing pathogens such as *P. gingivalis*, whose gingipains interact with ASN aggregates in Parkinson’s brains [100,155]. Oral–gut transmission enhances this process, as salivary phages alter downstream microbiota, perpetuating neurotoxic cascades [91,156].

### 7.4. Metabolic Biochemistry Linking Oral Microbiota to Dopaminergic and Serotonergic Signaling

The relationship between oral bacteria, their metabolic pathways, and neurotransmitter synthesis is an emerging research area. Like gut bacteria, oral microbes encode pathways that produce or degrade neurotransmitters such as dopamine, glutamate, tryptophan, and GABA, though in vivo biosynthesis remains to be investigated [125]. Tryptophan metabolism impacts serotonin synthesis, which is reduced in PD [157]. Oral microbial pathways are enriched in tyrosine metabolism and glutamate production, influencing brain reward circuits and overall function [2]. Notably, *Streptococcus mutans*-derived imidazole propionate promotes dopaminergic neuronal loss and motor deficits in PD models [158].

### 7.5. Dopamine Receptor-1 Regulation and Parkinson’s Disease Symptoms

The dopamine D1 receptor, found in the midbrain and forebrain, regulates motor control, reward, motivation, and cognition, with dysfunction linked to PD [159,160]. Changes in the gut microbiome influence dopaminergic signaling and DRD1 expression, such as through *Clostridioides difficile* infection [161], microbiome composition shifts associated with impulsivity [162]. Oral pathogens worsen neuroinflammation, promote dopaminergic neuron loss [138], and exacerbate PD motor and non-motor symptoms through microbiome-driven inflammation and neurotransmitter alterations [3].

## 8. Therapeutic Horizons

Therapeutic horizons in addressing oral microbial dysbiosis present promising avenues for mitigating neuropsychiatric disorders. For instance, the development of small-molecule inhibitors targeting gingipains offers a novel therapeutic strategy to ameliorate AD-related pathologies by reducing amyloid-beta plaque formation, tau tangles, and neuroinflammatory responses. Similarly, targeting other oral pathogens, such as *Aggregatibacter actinomycetemcomitans* and *Treponema denticola*, which provoke inflammatory responses and contribute to neuroinflammation by increasing cytokine production and amyloid-beta secretion, offers additional therapeutic avenues for modulating AD pathogenesis [3]. Given the presence of *P. gingivalis* in the brain and its direct association with AD pathologies, therapeutic interventions targeting gingipains could significantly reduce bacterial load, reduce neuronal inflammation, and prevent Aβ plaque formation [163]. Furthermore, blocking Cathepsin B and protein phosphatase 2A (PP2A), both implicated in neuroinflammation and tau hyperphosphorylation, could represent another effective therapeutic strategy for Alzheimer’s disease [97].

Beyond direct antimicrobial approaches, manipulating the oral microbiome through prebiotics, probiotics, and synbiotics may offer a promising strategy to restore microbial homeostasis and dampen systemic inflammation, thereby indirectly benefiting neurological health. These strategies aim to mitigate the systemic inflammatory burden originating from oral dysbiosis, thereby reducing its detrimental impact on the brain and overall neuropsychiatric well-being [2].

### 8.1. Probiotics

The Food and Agriculture Organization of the United Nations and the World Health Organization define probiotics as “live microorganisms which, when administered in adequate amounts, confer a health benefit on the host [164]. Probiotic microorganisms supply essential nutrients, facilitate host digestion of food, and compete with potential pathogens for adhesion sites and resources. Moreover, probiotics modulate the host immune system, inducing local and systemic responses that regulate inflammatory processes throughout the body [165]. This immunomodulatory potential, along with their direct antimicrobial activity against pathogens, establishes probiotics as a promising therapeutic strategy for diverse inflammatory conditions, including those associated with brain disorders [166] (Table 2).

### 8.2. Prebiotics and Synbiotics

Prebiotics are recognized as functional foods due to their significant role in promoting health and preventing disease [177,178]. These approaches collectively aim to counteract the “oralization” of the gut microbiota and subsequent translocation of toxic bacterial proteases to the brain, which contribute significantly to neuroinflammation and cognitive dysfunction. This intricate interplay between the oral and gut microbiomes underscores the critical importance of maintaining microbial homeostasis for systemic and neurological health, suggesting that therapeutic interventions targeting oral dysbiosis may offer significant neuroprotective benefits [3,179].

A synbiotic is defined by its composition, which includes a live microorganism and a selectively utilized substrate [180]. For synbiotics classified as complementary, their individual components must satisfy the requisite evidence and dosage criteria established for both probiotics and prebiotics [180]. Furthermore, the combined formulation must empirically demonstrate a health benefit in the target host, substantiated by an appropriately designed trial [180]. In addition to microbiome-focused interventions, growing attention has been directed toward phytogenic natural compounds as complementary strategies for AD. Preclinical evidence suggests that several plant-derived bioactive molecules can modulate neuroinflammatory signaling, particularly through the nuclear factor kappa B (NF-κB) pathway, acting in coordination with pathways such as Nrf2 and MAPK [181]. Although these approaches remain largely experimental, they underscore the therapeutic relevance of targeting shared inflammatory mechanisms across neurodegenerative disorders (Table 3).

## 9. Discussion

While multiple studies describe links between oral microbial changes and neuropsychiatric or neurodegenerative conditions, the available human evidence remains mainly observational and should be interpreted carefully [59,63,64,103]. Most clinical studies use cross-sectional or case–control designs, which are naturally limited to finding correlations and do not allow conclusions about causality or the order of events. Therefore, it is unclear whether changes in the oral microbiome happen before neurological disease, develop as a result of disease progression, or reflect shared risk factors such as age, medication use, systemic inflammation, or behavioral characteristics [59,67,103]. Additionally, epidemiological studies often depend on periodontal clinical indices or relative abundance data, which may not fully represent microbial function or pathogenic activity [103,186,187].

Methodological heterogeneity, including differences in sampling sites, sequencing approaches, and analytical pipelines, further limits comparability across studies and may contribute to inconsistent findings, particularly in anxiety and depression research [27,57,59,132]. Although experimental and animal studies provide biologically plausible mechanisms linking oral inflammation to neuroinflammatory processes, these models do not fully recapitulate the complexity of human disease and should not be interpreted as direct evidence of causation in clinical populations [29,66]. Importantly, the variation in study designs and laboratory workflows largely explains the apparent inconsistencies in the literature. In humans, most datasets are cross-sectional or case–control, so reported differences should be seen as associations rather than evidence of temporal or causal relationships, especially since neurological outcomes can influence oral hygiene, diet, medication exposure, and systemic inflammation. Additionally, across studies, the source of oral samples (e.g., saliva versus site-specific plaque), sequencing methods, and bioinformatic pipelines vary greatly, which limits comparability and may explain why some taxa seem enriched in disease in one cohort but not in another. Many clinical reports also depend on periodontal indices or relative abundance profiles, which may not reflect microbial function or pathogenic activity, further complicating synthesis when results conflict at the taxonomic level. Lastly, while animal and experimental models support biological plausibility, they do not fully replicate the complexity of human neurodegenerative or neuropsychiatric conditions; therefore, mechanistic findings should be considered supportive rather than conclusive clinical evidence.

The evolving concept of the oral–brain axis signifies a fundamental reorientation in our understanding of neuropsychiatric pathologies. Dysbiosis of the oral microbiome acts not merely as a localized factor in oral disease but also as a systemic driver of neuroinflammation and central nervous system impairment. This association resembles the more widely recognized gut–brain axis; however, it provides a distinct pathway for the entry of microbial and inflammatory agents directly connected to cranial nerves, the circulatory system, and immune signaling networks [2,15,55].

Therapeutically, the oral–brain axis offers promising avenues. Periodontal treatment has been shown to reduce systemic cytokine levels, supporting the plausibility of modifying the neuroinflammatory burden through oral interventions [186]. In addition, probiotics, prebiotics, and synbiotics show growing potential as modulators of the oral microbiome with systemic immunomodulatory effects [175,178,188]. Yet, variability in strain selection, dosing, and outcome measurement underscores the need for standardized clinical protocols. Future research should prioritize longitudinal human studies evaluating temporal relationships between dysbiosis and neurological outcomes; multi-omics approaches integrating metagenomics, metabolomics, and immunophenotyping; randomized clinical trials assessing periodontal and microbiome-focused therapies; and the development of oral biomarkers for early detection of neurodegenerative disease. Given the accessibility of the oral cavity, such biomarkers could significantly advance early diagnosis and precision prevention.

Given the bidirectional microbial transmission between the oral cavity and the gastrointestinal tract, interventions that rebalance the oral microbiota can also positively impact the gut microbiome, further enhancing systemic health and mitigating neurological disorders [95]. These therapeutic strategies, spanning targeted molecular interventions to comprehensive dietary modifications, underscore the importance of a holistic approach to managing oral health for neurological benefit. Further exploration of these integrated approaches is warranted to elucidate their efficacy in clinical settings fully and to establish standardized protocols for patient care [3]. Future research should therefore prioritize comprehensive studies that evaluate the long-term impact of oral microbiome-modulating therapies on neuropsychiatric outcomes, while also investigating personalized approaches based on an individual’s unique microbial signature and genetic predispositions [3,189].

In summary, the oral microbiome represents a modifiable factor with profound implications for neuroinflammation and CNS health. Integrating oral health into neurological and psychiatric research frameworks may open the way to new preventive and therapeutic strategies that could reduce the global burden of brain disorders. Overall, the current literature supports a potential association between oral dysbiosis and neuroinflammatory pathways but remains insufficient to establish a causal relationship in humans. Carefully designed longitudinal studies, standardized methodologies, and interventional trials are required to clarify these relationships and assess the clinical relevance of oral microbiome modulation [61,103,186].

## 10. Conclusions

The evidence compiled in this review highlights the oral microbiome as a significant yet understudied factor in the pathogenesis of neuropsychiatric and neurodegenerative disorders. Oral dysbiosis, especially in chronic periodontal disease, promotes systemic inflammation and microbial dissemination, contributing to disruption of the blood–brain barrier and neuroinflammatory processes. Collectively, findings across Alzheimer’s, Parkinson’s, and mood disorders suggest a shared pathway in which microbial and immune interactions impair neuronal stability. Integrating oral health management into neurological prevention through periodontal care, microbiome modulation, and anti-inflammatory strategies may represent a promising approach for early intervention. Future research should aim to establish causal links and evaluate whether restoring oral microbial balance can modify disease progression.

## Figures and Tables

**Table 1:** PRISMA-like overview of literature identification and selection (Narrative Review).

Stage	Description
**Type of review**	Narrative review (conceptual and mechanistic synthesis; not a systematic review)
**Databases searched**	PubMed/MEDLINE, Scopus, Web of Science, Google Scholar
**Timeframe of literature considered**	No a priori lower date limit was imposed. The review integrates **foundational studies and contemporary literature**, with cited references spanning approximately **2015 to 2025**, reflecting both seminal mechanistic evidence and recent advances in oral microbiome and neuroinflammation research
**Search focus/key concepts**	Oral microbiome dysbiosis; periodontal pathogens; oral–brain axis; oral–gut–brain axis; neuroinflammation; cytokine signaling; blood–brain barrier disruption; Alzheimer’s disease; Parkinson’s disease; anxiety; depression
**Study types considered**	Human observational studies (cross-sectional, case–control, epidemiological); experimental and animal models; molecular and mechanistic studies
**Inclusion approach**	Studies were included based on relevance to oral microbiome alterations and their mechanistic or associative links to neuropsychiatric and neurodegenerative disorders
**Exclusion approach**	Articles not addressing oral microbiome, oral pathogens, or neuroinflammatory/neurobiological outcomes were not considered central to the narrative synthesis
**Screening strategy**	Iterative, topic-driven screening conducted to support conceptual integration rather than exhaustive quantitative selection
**Data extraction**	Qualitative synthesis of immune, metabolic, neural, and microbial virulence mechanisms, as well as therapeutic implications
**Assessment of bias/quality**	Formal risk-of-bias assessment not performed; methodological limitations and heterogeneity are explicitly discussed in the Discussion section

**Table 2:** Probiotics.

Probiotic Strain	Native Location	Mechanism in the Oral Cavity	Oral Microbial Effects
***Streptococcus oralis KJ3***	Teeth, gingiva	Produce hydrogen peroxide (H_2_O_2_) via pyruvate oxidase (SpxB). H_2_O_2_ inhibits competing bacteria, particularly anaerobic and acidogenic species associated with caries and periodontal disease. In addition, *S. oralis* produces bacteriocin-like inhibitory substances that suppress closely related streptococci, contributing to microbial balance within oral biofilms.	Limits adhesion and overgrowth of cariogenic and periodontopathogenic species, including *Streptococcus mutans* and *Porphyromonas gingivalis* [167,168,169]
***Streptococcus uberis KJ2***	Teeth, gingiva	Hydrogen peroxide production	Inhibition of oral pathogens involved in gingivitis and periodontitis [167,168]
***Streptococcus ratus JH145***	Teeth	lactate dehydrogenase (LDH) deficient, competitive exclusion	Inhibition and competitive exclusion of oral pathogens involved in caries [168,170]
***Streptococcus salivarius BLIS M18***	Dorsum of the tongue and the pharyngeal mucosa	Bacteriocin production (lantibiotics salivaricin A2, salivaricin 9, salivaricin M) contains the arginine deiminase system (ADS), which converts arginine to ammonia and raises local pH	Reduce oral pathogens implicated in gingivitis, periodontitis, and caries. Decrease the levels of volatile sulfur compounds involved in halitosis [171,172]
***Bifidobacterium*** **spp.**	Teeth, saliva, tongue dorsum	*Bifidobacterium* species can adhere to oral epithelial cells, dental surfaces, and salivary pellicle components via surface proteins, pili, and exopolysaccharides. *Bifidobacterium* produces organic acids (mainly acetic and lactic acid), bacteriocin-like substances, and other antimicrobial metabolites that inhibit oral pathogens.	Competition with oral pathogens for binding sites, reducing colonization by cariogenic and periodontopathogenic bacteria such as *Streptococcus mutans* and *Porphyromonas gingivalis* [169,173,174].
***Lactobacillus*** **spp.**	Teeth, saliva, tongue dorsum	*Lactobacillus* species can compete with oral pathogens for adhesion sites on mucosal surfaces and dental tissues through hydrophobicity and specific adhesins. Oral *Lactobacillus* strains produce organic acids (lactic, acetic), hydrogen peroxide, bacteriocins, and strain-specific metabolites	These compounds directly inhibit the growth of *Streptococcus mutans*, *Porphyromonas gingivalis*, *Candida* spp., and other oral pathogens [168,174,175,176].

**Table 3:** Prebiotics and symbiotics.

**Prebiotic**		
**Compound**	**Mechanism in the Oral Cavity**	**Oral Microbial Effects**
**L-arginine**	Arginine is utilized by arginolytic oral bacteria through the arginine deiminase system (ADS), leading to ammonia generation and a consequent increase in local biofilm pH. In addition, this pathway influences biofilm matrix formation and can modify the expression of genes associated with microbial virulence.	This mechanism elevates biofilm pH, diminishes the ecological competitiveness of acidogenic and aciduric cariogenic bacteria (such as *S. mutans*), and favors the growth or metabolic activity of health-associated streptococci possessing the arginine deiminase system [182].
**Xylitol**	As a non-fermentable sugar alcohol for *S. mutans*, this compound disrupts bacterial carbohydrate uptake and metabolism through energy-wasting cycles, leading to reduced adhesion and lower mutans streptococci levels. When administered via chewing gum, it also stimulates salivary flow, enhancing mechanical clearance.	It is associated with a selective decrease in mutans streptococci in dental plaque and saliva, attenuation of cariogenic potential, and reductions in caries incidence or mutans counts with regular exposure, depending on dose and delivery method [183].
**Dietary Nitrate (NO_3_^−^)**	Acts as a substrate for oral nitrate-reducing bacteria, which convert nitrate to nitrite and subsequently to nitric oxide (NO) and other reactive nitrogen species. These metabolites exhibit antimicrobial and immunomodulatory activities and can influence microbial metabolic pathways.	Decrease in selected periodontopathogens and certain cariogenic genera, and modulation of biofilm metabolism and local pH toward a more homeostatic state. Additional benefits may include anti-inflammatory effects mediated by NO [184].
**Inulyn type fructans (FOS)**	Act as non-sucrose carbohydrate substrates that alter competitive interactions within oral biofilms. They do not selectively target pathogens but instead influence community structure and metabolic balance, consistent with the ecological plaque hypothesis	Formation of less virulent, less adhesive biofilms compared with sucrose-driven biofilms [175].
**Galacto-oligosaccharides (GOS)**	Display higher selectivity for bifidobacteria and other health-associated microbes. GOS reduce prolonged low-pH episodes and indirectly suppress the ecological conditions that favor caries development	Promotion of a more stable and health-associated biofilm composition [175].
**Synbiotics**		
**Formulations**	**Mechanism in the oral cavity**	**Oral Microbial Effects**
***Lactobacillus rhamnosus*** **GG + Inulin/FOS**	This synbiotic combination improves competitive exclusion of *Streptococcus mutans* through nutrient competition and biofilm interference, without promoting EPS-rich cariogenic biofilms.	Formation of less adhesive and less virulent biofilms [173].
***Lactobacillus reuteri*** **(DSM 17,938/ATCC PTA 5289) + Inulin-type fructans**	Inulin enhances the survival and functional activity of *Lactobacillus reuteri*, thereby promoting the synthesis of antimicrobial metabolites, including reuterin and reutericyclin, which exhibit inhibitory effects against periodontal pathogens.	Decreased levels of *Porphyromonas gingivalis* and *Aggregatibacter actinomycetemcomitans* [133].
***Bifidobacterium animalis subsp. lactis + GOS***	Galacto-oligosaccharides preferentially promote bifidobacterial metabolic activity while being inefficient substrates for cariogenic streptococci. This selective utilization supports the ecological competitiveness of Bifidobacterium within multispecies oral biofilms and mitigates acidogenic predominance.	Reduced biofilm acidogenicity and Improved pH recovery after carbohydrate exposure [175,185].
***Lactobacillus rhamnosus*** **GG + Inulin/FOS**	This synbiotic combination improves competitive exclusion of *Streptococcus mutans* through nutrient competition and biofilm interference, without promoting EPS-rich cariogenic biofilms.	Formation of less adhesive and less virulent biofilms [173].

## Data Availability

The original contributions presented in this study are included in the article. Further inquiries can be directed to the corresponding authors.
